# The Effect of Thermal Annealing on Optical Properties and Surface Morphology of a Polymer: Fullerene- and Non-Fullerene-Blend Films Used in Organic Solar Cells

**DOI:** 10.3390/polym18020280

**Published:** 2026-01-20

**Authors:** Bożena Jarząbek, Muhammad Raheel Khan, Barbara Hajduk, Andrzej Marcinkowski, Paweł Chaber, Adrian Cernescu, Yasin C. Durmaz

**Affiliations:** 1 Centre of Polymer and Carbon Materials, Polish Academy of Sciences, Sklodowskai-Curie 34 Str., 41-819 Zabrze, Poland; bhajduk@cmpw-pan.pl (B.H.); amarcinkowski@cmpw-pan.pl (A.M.); pchaber@cmpw-pan.pl (P.C.); 2 Joint Doctoral School, Silesian University of Technology, Akademicka 2A, 44-100 Gliwice, Poland; 3 Attocube systems AG, Eglfinger Weg 2, 85540 Munich, Germany; adrian.cernescu@attocube.com (A.C.); yasin.durmaz@attocube.com (Y.C.D.)

**Keywords:** donor–acceptor blend films, PTB7-Th, PC70BM, ZY-4Cl, UV-VIS-NIR and ellipsometric analysis, AFM and Nano-IR studies, needle-shape crystallites

## Abstract

The optical properties, electronic structure and morphology of thin films of the polymer donor PTB7-Th blended with either the fullerene acceptor PC70BM or the non-fullerene acceptor ZY-4Cl were systematically investigated to evaluate their annealing-induced evolution. Thin films were characterized using UV–Vis–NIR absorption spectroscopy, spectroscopic ellipsometry, ATR-FTIR spectroscopy, atomic force microscopy (AFM), and nano-IR analysis. In situ stepwise thermal annealing revealed distinct changes in absorption edge parameters, indicating thermally induced modifications in the electronic structure of the blend films. Ellipsometric analysis showed that elevated temperatures significantly affect the refractive index and extinction coefficient spectra. AFM measurements demonstrated markedly different surface morphology evolution for the two blend systems, with pronounced needle-shaped crystallites formation observed in PTB7-Th:ZY-4Cl films after annealing at 100 °C. Nano-IR characterization identified these crystallites as predominantly PTB7-Th, indicating phase separation driven by thermal treatment. The combined optical and structural results reveal distinct annealing-induced changes in the blend. Finally, BHJ solar cells, based on PTB7-Th:PC70BM and PTB7-Th:ZY-4Cl active layers, were fabricated, and their photovoltaic response was demonstrated.

## 1. Introduction

Solar energy, as an abundant and sustainable resource, has gained significant attention in recent decades [1]. Traditional inorganic semiconductor solar cells, such as those based on silicon (Si) and gallium arsenide (GaAs), are well-known for their high efficiency and broad light absorption capabilities. However, their widespread deployment is hindered by high production costs associated with the complex fabrication process, including high temperature treatments [2,3]. Organic solar cells (OSCs) have attracted significant attention from both academia and industry due to their low production cost, light weight, flexibility and semi-transparency [4,5,6,7]. In organic photovoltaic devices, the predominant advancements in power conversion efficiency (PCE) are attributed to the molecular engineering of photoactive materials and the strategic optimization of active layer morphologies and architectures [8,9,10,11,12,13,14]. The bulk heterojunction (BHJ) active layer, composed of donor and acceptor materials, is mostly commonly used in OSCs, which allows for the improvement of charge separation and transport [15,16,17]. For optimal active layer morphology, the crystal domain size should be in the range of (10–20 nm), with high phase purity, vertical phase separation and crystalline interpenetrating network [18]. Controlling the morphology of active layers is challenging due to the different crystallization behaviors of donor and acceptor materials. To date, various optimization methods and post-deposition strategies have been explored to achieve improved morphology and enhanced photovoltaic performance [19].

The optimization process of the BHJ active layer can be achieved through post deposition treatments, including solvent vapor annealing, thermal annealing and/or introducing a solid additive into the blend. These approaches promote molecular reorganization, improve phase separation and enhance crystallinity, ultimately leading to better device performance [20,21,22]. The solvent vapor annealing (SVA) is also an effective approach for controlling the morphology of the active layer, wherein the blend film is exposed to the vapor of an annealing solvent, particularly at room temperature and/or at elevated temperature. In order to optimize this process, both the annealing temperature, time and saturated vapor pressure are important factors [23,24]. Nowadays, solvent additive is an effective method to control the morphology of active layers. Generally, a high-boiling-point solid additive is incorporated into the blend to tune the crystallinity and phase separation. However, the drawback of this higher boiling point of the solvent additive is that it remains in the active layer, which affects the photo-stability of OSCs. Hence, the solid additive has been developed in order to minimize the undesirable effect of the solvent additive [25,26,27].

Fullerene (FA) and non-fullerene (NFA) are the two types of acceptor materials used in BHJ active layers in organic solar cells. Currently, non-fullerene-type acceptors are widely used due to their superior properties. However, the benefits of fullerene acceptors cannot be ignored due to the high electron affinity and mobility at room temperature, i.e., 1 cm^2^ V^−1^S^−1^. At the same time, rather high preparation costs, poor time-stability and weak absorption can limit the application of these FA materials [28,29,30,31]. Non-fullerene acceptors offer potential advantages such as reduced voltage losses and lower production cost; moreover, their energy level can be easily tuned [28,29,30,31,32].

In the first generation of OSCs, the single active layer was sandwiched between two electrodes with different work functions. The single-layer devices showed poor efficiency, below 0.1%, due to the lack of efficient dissociation of excitons (electrons and holes) [33]. A bilayer structure introduced by Tang [34], consisting of copper phthalocianine, as a donor and perylene tetracarboxylic derivative as an acceptor, achieved of 1% PCE. The limited PCE of bilayer organic solar cells (OSCs) is primarily attributed to inefficient exciton diffusion and separation at the donor–acceptor (D/A) interface [35]. To address this, Yu et al. [36] introduced bulk heterojunction (BHJ) blend films, where donor and acceptor materials are intermixed to form a single active layer. This architecture enhanced OSC performance by reducing the exciton diffusion distance [36]. The development of BHJ OSCs marked a significant breakthrough in organic electronics, with reported PCE values reaching up to 19.3% [37].

Post-deposition treatment is an important factor for achieving the higher fill factor (FF) and power conversion efficiency (PCE), and is also important for molecular ordering. Device optimization is the main strategy to achieve highly efficient solar cells. Mendez et al. [38] studied the influence of post-deposition treatment on the DPP(TBFu)_2_:PC61BM active layer system, showing that intra- or inter-molecular interaction form well-defined crystal domain size. Molecular ordering enhanced charge transport, reduced charge recombination and increased the D/A phase separation. Processing conditions have a major impact on the BHJ morphology and on the optoelectronic performances [39]. Güney et al. [40] investigated the performance of P3HT:PCBM-based solar cells, annealed at different temperatures (80–160 °C), and compared them with non-annealed devices. They concluded that 120 °C is the optimal annealing temperature, as both the external quantum efficiency (EQE) and device stability were significantly enhanced at this temperature. Zuo et al. [41] investigated the small molecule (DRDTSBDTT) in order to know the impact of annealing on current density–voltage (J-V) characteristics and active layer morphology. They demonstrated a strong correlation between annealing conditions and J-V behavior and showed that morphology optimization and phase control are critical for achieving high-performance organic solar cells. In [42], the effects of high-temperature annealing (up to 290 °C) on the properties of PTB7 and PTB7:fullerene blends were investigated. However, such thermal treatment improved morphology; it degraded optoelectronic properties due to side chain cleavage, which produces by-products that act as trap states, thereby increasing electronic disorder and reducing charge carrier mobility [42]. Generally, solar cells are usually exposed to the higher “working” temperatures (often above 25 °C), so it is important to know and understand their behavior during annealing. In this work, we present a systematic study on the influence of acceptor type (fullerene or non-fullerene) on the properties of donor–acceptor (D-A) blend films at higher temperatures, as well as on the efficiency and key photovoltaic parameters of BHJ solar cells, based on these active layers.

In this paper, PTB7-Th:PC70BM and PTB7-Th:ZY-4Cl blend films were prepared by spin coating, and various experimental techniques were employed to assess their thermo-stability. Additionally, to compare the role of individual ingredients of blends, the results obtained for pristine PTB7-Th, ZY-4Cl, and PC70BM films are presented in the Supplementary Information (SI). UV-Vis-NIR(*T*) absorption spectra of the blend films were recorded in situ during the stepwise, controlled annealing. The temperature dependence on absorption edge parameters, i.e., the energy gap *E*_G_ = *f*(*T*) and the Urbach energy *E*_U_ = *f*(*T*) were determined, presented and discussed in this work. A similar investigative approach was previously applied by us to the P3HT:PCBM (1:1) blend, as well as to pristine P3HT and PCBM films [43] to compare the thermo-optical behavior of neat material films with the properties of polymer–fullerene blended films. Furthermore, the effects of the annealing process and iodine content (0, 1, 5 and 10 mol.%) on iodine (I_2_)-doped pristine P3HT and P3HT:PCBM blend films were investigated using UV-Vis-NIR(*T*) and X-ray diffraction methods [44]. The temperature associated with iodine release from the doped blend films was determined, and the thermally inducted structural changes in both the polymer and blend films were analyzed [44]. Other experimental methods were applied by us to investigate the effect of chemical structure on thermal, optical and electrochemical properties of conjugated polyazomethine thin films [45].

Herein, changes in the refractive index and exciton coefficient for both types of blend films, after annealing at 100 °C and 200 °C, were presented, using the ellipsometric measurements. The role of the acceptor type (FA or NFA) within the blend film after annealing was clearly seen on the AFM images. To explain the spectacular changes in surface morphology, we employed a specialized nano-IR method in which AFM-based scattering-type scanning near-field optical microscopy (s-SNOM) was integrated with a broadband illumination source. This approach enabled the acquisition of material-specific maps of chemical and optical properties simultaneously with the topographic AFM imaging of the film surface. In the case of the needle-shaped crystallites seen on the non-fullerene (NFA)-based blend film surface after annealing, we could identify the type of crystallite material as mainly PTB7-Th.

The main objective of this work was to present a comprehensive study of the changes in electronic structure and surface morphology during these blend films in thermal annealing, with the aim of identifying the most suitable annealing temperature for the post-deposition strategy and to understand the role of temperature and type of acceptor within these D-A blend films. Finally, the BHJ organic solar cell devices based on these active layers (with FA or NFA) were fabricated, and the photovoltaic parameters of both device types were determined.

## 2. Experiment

### 2.1. Materials

**PTB7-Th:** Poly[4,8-bis(5-(2-ethylhexyl)thiophen-2-yl)benzo[1,2-b;4,5-b′]dithiophene-2,6-diyl-alt-(4-(2-ethylhexyl)-3-fluorothieno[3,4-b]thiophene-)-2-carboxylate-2-6-diyl] (Mw 133.198, Ossila Ltd., Leiden, The Netherland) was used as a donor material in the BHJ structure.

**ZY4Cl** is a modified version of BTP-4Cl-12 and belongs to the Y6 non-fullerene acceptors family with a fused thienothienopyrrolo-thienothienoindole (TTP-TTI) core and 5,6-dichloro-1H-indene-1,3(2H)-dione peripheral end groups: 2,2′-((2Z,2′Z)-((12,13-bis(2-butyloctyl)-3,9-diundecyl-12,13-dihydro-[1,2,5]-thiadiazolo[3,4-e]thieno[2″,3″:4′,5′]thieno[2′,3′:4,5]pyrrolo[3,2-g]thieno[2′,3′:4,5]thieno[3,2-b]indole-2,10-diyl)bis(methane-lylidene)))bis(5,6-dichloro-1H-indene-1,3(2H)-*dione*) (>98 wt% purity, Mw 1533.87, Ossila Ltd., Leiden, The Netherland).

**PC70BM** is a fullerene acceptor ([6,6]-Phenyl-C71-butyric acid methyl ester (>99 wt% purity, Ossila Ltd., Leiden, Netherland), and chlorobenzene purchased from WARCHEM (Poland) were used as the solvent, with all materials used as received.

**Figure 1:**
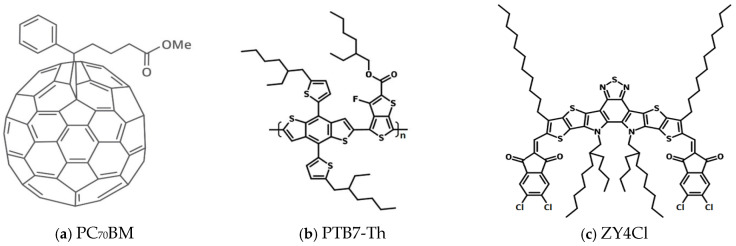
Chemical structure of (**a**) PC70BM, (**b**) PTB7-Th and (**c**) ZY-4Cl.

The chemical structures of the used materials are shown in Figure 1a–c.

### 2.2. Thin Films Deposition Method

Solutions in chlorobenzene were prepared at a concentration of 10 mg/mL, and the mixed solutions were stirred for 24 h at room temperature. Substrates, including quartz, microscopic glasses and silicon glasses, were sequentially cleaned using deionized water and isopropanol, each for ten minutes in an ultrasonic bath. Thin films were deposited onto the quartz, microscopic and silicon substrates by spin coating at 1000 rpm. The film thickness, measured by ellipsometry and AFM, was approximately 100 nm (±25 nm).

### 2.3. Measurement Techniques

- UV-Vis absorption

Optical measurements were carried out through a two-beam UV-Vis-NIR (200–2500 nm) JASCO V-570 (JASCO Co., Ltd., Tokyo, Japan) spectrophotometer, working with the special Spectra Manager software (V-570/C0296872). Transmission (*T*%) and fundamental reflectivity (*R*%) of thin films on quartz substrates were registered at room temperature. During the reflectivity measurements, a special two-beam reflectance arrangement was used, with an Al mirror in the reference beam, as a reflectance standard. Then, blend films were exposed to the stepwise annealing, using a special auto-control equipment of the JASCO spectrometer, where transmission can be registered in situ at a specific, defined temperature. The special in situ computer program was used to control the rate of heating and the temperature of the sample. Transmission spectra were recorded within the range of 25 °C to 200 °C, for every 25 °C, and the temperature was gradually increased at a rate of 5 °C/min. After the last step at 200 °C, the samples were cooled to room temperature, and the spectra were recorded.

- Ellipsometric measurements

**Figure 2:**
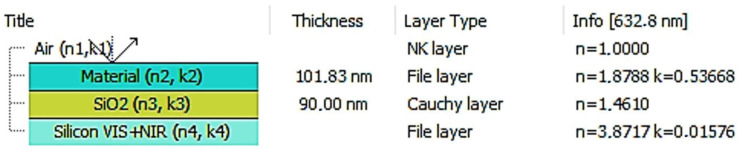
The applied ellipsometrical model.

Spectroscopic ellipsometer SENTECH SE850E (SENTECH Instruments GmbH., Berlin, Germany), working within the 240–2500 nm wavelength range, equipped with a variable-temperature cell, together with the optical microscope (enlargement ×10) were used to obtain the surface images (the scan area 1 mm in diameter) during annealing/cooling runs. This device is operated by the Spectra Ray-3 software, and the optical results of samples were obtained using a variable-angle spectroscopic ellipsometry (VASE), in the angle range of 40–70 °C; every measurement was taken with an incremental step of 10 °C. The optical system, in our case, consists of four layers and is presented in Figure 2.

Polymers and blend films were deposited onto silicon substrates, covered with ~90 nm silicon dioxide. The silicon layer was fitted using a file layer, while the silicon dioxide layer was modeled with a Cauchy layer. Other materials were also fitted using the file layer approach. For the polymeric film model layers, ellipsometric angles were fitted using point-by-point approximation to achieve the lowest possible mean square error (MSE) value.

The first step involved measuring clean substrates, followed by ellipsometric fitting of the substrate model. In the next step, the ellipsometric angles *Ψ* and *Δ* were fitted to the films deposited on these substrates.

- AFM method

Atomic force microscopy (AFM) images and the thicknesses of the films were obtained by using a dimension ICON AFM, which is equipped with a nano-scope V controller (Bruker Corporation, Santa Barbara, CA, USA) operating in the soft tapping mode, with a scan rate of 1 HZ in an air atmosphere, with a standard tip 125 µm long. Images were obtained with a piezoelectric scanner with a nominal size of (85 × 85) µm. Nano-scope Analysis 1.9 software (Bruker corporation, Santa Barbara, CA, USA) was used to record micrographs.

- ATR-FTIR studies

Fourier transform infrared (FTIR) spectra were recorded using a JASCO FT/IR-6700 (JASCO Co., Ltd., Tokyo, Japan) spectrometer, which was equipped with an ATR attachment, containing a single-reflection diamond crystal (ATR PRO670H-S). The infrared spectra were measured with a resolution of 4 cm^−1^ over a spectral range of 400–4000 cm^−1^. A total of 32 scans were accumulated for each sample. These spectra were recorded as follows: 10 µL of chlorobenzene-based solutions were transferred to the ATR crystal, and the spectra were measured once chlorobenzene evaporated.

- Nano-IR characterization

Nano-FTIR measurements were conducted using a nea-SCOPE system (commercially available from attocube.com/neaspec), which integrates an atomic force microscopy (AFM)-based scattering-type scanning near-field optical microscopy (s-SNOM) with a broadband illumination source. A platinum–iridium-coated metallic tip is illuminated by a coherent difference frequency generation laser (Toptica Photonica AG, Gräfelfing, Germany) that spans a spectral range from 600 to 2200 cm^−1^ and delivers ca. 500 µW of power. The back-scattered light resulting from the tip-sample interaction is detected interferometrically, using an asymmetric Michelson interferometer, at a liquid nitrogen-cooled mercury–cadmium–telluride (MCT) detector. This configuration enables the extraction of complex phase and amplitude values corresponding to vibrational absorption and reflection. The probe tip is oscillated at its fundamental resonance frequency (Ω) and demodulated at higher harmonics (nΩ) to minimize background contributions, effectively isolating the near-field signal that encodes local material information. Complementary nanoscale images were also acquired using a similar microscope setup, incorporating a tunable quantum cascade laser (QCL) and pseudo-heterodyne (Ps-Het) detection at selected wavelengths.

- Photovoltaic cells—preparation and characterization

The photovoltaic devices were fabricated on commercially available Ossila company ITO-coated glass substrates with dimensions of 20 mm × 15 mm. An 8-pixel shadow mask was used during the deposition of the top electrode to define the device geometry, where each pixel corresponds to an active area of 0.04 cm^2^. Consequently, eight electrically independent bulk-heterojunction (BHJ) photovoltaic devices were fabricated on a single substrate under identical processing conditions. Prior to device fabrication, the ITO-coated substrates were cleaned with deionized water and isopropanol in the ultrasonic bath. A thin film of PEDOT:PSS was deposited on the cleaned ITO substrate by spin coating at a spin speed of 5000 rpm and then annealed at 110 °C for 10 min in air. Active layer solutions were prepared by dissolving PTB7-Th:PC70BM and PTB7-Th:ZY4Cl (1:1 wt.) in chlorobenzene. The prepared solutions were spin coated, with a spin speed of 1000 rpm, on the PEDOT:PSS (as the BHJ active layers, i.e.,PTB7-Th:PC70BM and PTB7-Th:ZY-4Cl) and then annealed at 120 °C and 100 °C, respectively, for the optimal morphology formation. Subsequently, an aluminum (Al) back contact electrode was evaporated on top of the blend thin film.

The current density–voltage (J–V) characteristics of the prepared photovoltaic devices were measured using a solar simulator under 1000 W/m^2^ illumination, with a Keithley 2400 Source Meter (SMU) instrument (Tekronix, Poland).

## 3. Results and Discussion

### 3.1. Temperature Dependence of Absorption Spectra of Thin Films

Transmission (*T*) spectra of thin films of PTB7-Th:PC70BM and PTB7-Th:ZY-4Cl (wt. ratio 1:1) blends have been recorded in situ, during annealing runs, at every 25 °C, in a temperature range from 25 to 200 °C. The absorption coefficient (*α*) vs. photon energy (*E*) plots of the thin films, at each investigated temperature, were obtained using a simple equation [43]:

(1) $$\alpha =\frac{1}{d}\ln\left(\frac{1}{T}\right)$$

**Figure 3:**
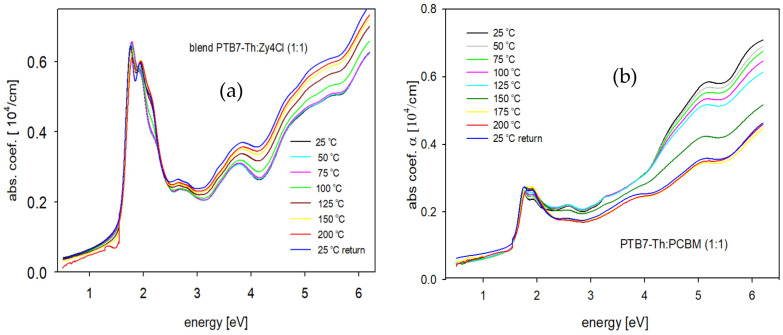
Absorption coefficient spectra, obtained during in situ annealing blend films on quartz substrates: (**a**) PTB7-Th:ZY-4Cl and (**b**) PTB7-Th:PC70BM.

Neglecting the reflectivity (*R*) due to its low level (about 5–8%) for each film. The thickness (*d*) of thin films changed after the annealing/cooling run (by 10%); however, for calculation purposes, it was assumed that the thickness of each thin film was constant during the experiment. This assumption was made due to the negligible thickness changes and also due to the impossibility of indicating the exact moments when the thickness had been changing (it was measured before and after the annealing/cooling run). Figure 3 presents these dependencies for both types of blends. While the absorption coefficient spectra of thin films of PTB7-Th and ZY-4Cl obtained at room temperature and then after annealing at 100, 150, and 200 °C are shown in the Supplementary Information (SI), as Figure S1, and for the pure PC70BM thin film, the temperature dependence of the absorption coefficient is presented in [43].

**Figure 4:**
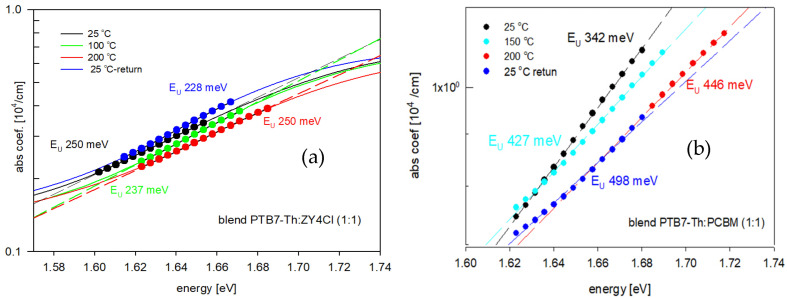
The Urbach energies of the investigated blends for chosen temperatures: (**a**) PTB7-Th:ZY-4Cl and (**b**) PTB7-Th:PC70BM.

**Figure 5:**
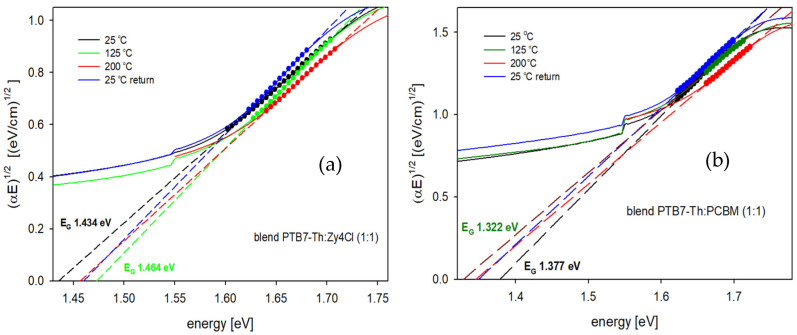
Tauc dependence used to obtain energy gaps for blends at chosen temperatures: (**a**) PTB7-Th:ZY-4Cl and (**b**) PTB7-Th:PC70BM.

Generally, the edge of absorption, being the low-energy wing of the first low-energy absorption band (the π → π* transition band of investigated films), can be subjected to a more detailed analysis, that is, the designation of absorption edge parameters: the energy gap width (*E*_G_) and the Urbach energy (*E*_U_). Methods used to obtain both parameters for the investigated blends are shown in Figure 4 and Figure 5. The value of the energy gap of a conjugated polymer depends on the length of conjugation in the polymer chain, while that of the Urbach energy is connected with the localized, defect states within the energy gap. The absorption edges of the investigated thin films exhibit an exponential region, which can be described by the Urbach relation [46]:

(2) $$\alpha \propto exp\left(\frac{E}{E_{U}}\right)$$

The Urbach energy is a “width of the band tail”, occurring due to localized states within the energy gap, caused by possible structural defects, like a break, torsion or aberration of polymer chains or molecules [47], hence the “Urbach–like” behavior of absorption edges of investigated films. The *E*_U_ values of thin films were calculated based on the slope of the exponential edge, following the Urbach equation, using the semi-logarithmic plots, as seen in Figure 4, for chosen temperatures.

The energy gaps of the investigated films were obtained (see Figure 5) based on the linear approximation to the energy axis, of the following relation [48]:

(3) $$\alpha \propto \;{\left(E-E_{G}\right)}^{2}\;\mathrm{for}\;(E\;>E_{G})$$

This dependence, known as the Tauc relation, is typical for amorphous semiconductors, often used also for amorphous polymers [43,44]. Since the X-Ray diffraction patterns for all the investigated films before and after the annealing/cooling process did not show any distinct sharp peaks, this dependence was used. Both the *E*_U_ and *E*_G_ values of the investigated films were calculated for each of the applied temperatures.

**Figure 6:**
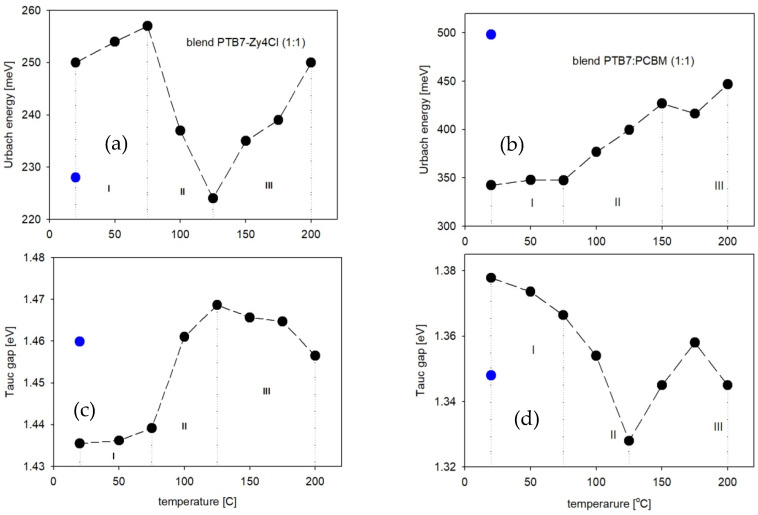
Absorption edge parameters of blend films PTB7-Th:ZY4Cl (**a**,**c**) and PTB7-Th:PCBM (**b**,**d**) during in situ annealing (black points) and after cooling to room temperature (blue points).

The most characteristic changes in the blend absorption edge parameters during annealing are presented below in Figure 6, and it is clearly seen that this quite different behavior of the Urbach energy and Tauc gap, and of blend films at higher temperatures, depends on the type of acceptor. It means that the type of material used as an acceptor can change the properties of blend films with the same donor material.

The temperature dependences presented in Figure 6 differ significantly for the two types of blends containing different acceptors. In particular, the variations in the Urbach energy and energy gap values for the PTB7-Th:PC70BM (1:1) blend film are similar to those reported in [43] for the P3HT:PCBM (1:1) blend film, where the same fullerene acceptor was used. Thermo-optical studies on the pristine PCBM thin film demonstrated its stability; both the absorption spectra and absorption edge parameters turned out to be unchanging during annealing/cooling runs [43]. In our present studies, we decided to investigate the absorption coefficient spectra and edge parameters at room temperature and after annealing at 100, 150 and 200 °C, respectively, for the pristine films of ZY-4Cl (acceptor) and PTB7-Th (donor) as depicted in Figure S1 in the Supplementary Information (SI) to discuss their role in blends, during annealing. When we compare dependences in Figure 6 and Figure S1, it is shown that in the case of the blend with the non-fullerene acceptor (ZY-4Cl), this compound decides on the blend behavior at higher temperatures, while for the blend with the fullerene acceptor, the donor PTB7-Th plays the main role because PC70BM is known for its thermal stability, and the absorption edge parameters of PC70BM film are almost constant during annealing [43]. The analysis of the temperature dependences of the Urbach energy and Tauc gap, as shown in Figure 6, can be divided into three distinctive stages.

(I) 25–75 °C—the changes in absorption edge parameters are rather insignificant for both types of blends.

(II) 75–125 °C—we can observe fairly rapid changes, but these are quite different for each type of blend. For the PTB7-Th:ZY-4Cl blend, the Urbach energy decreases from 257 meV to 224 meV, and the energy gap increases from 1.439 eV to 1.469 eV. These tendencies show that it is possible that the thermal reduction in structural defects, together with the worse length of conjugation. The similar dependences are seen for the pristine ZY-4Cl film (Figure S1a). In the case of the blend with fullerene, the opposite temperature dependences are seen. The Urbach energy increases from 347.5 meV at 75 °C to 426.9 meV at 150 °C, which can be related to the thermally induced disorder and due to the initiation of phase separation, particularly after exceeding the glass transition temperature of PCBM (*T*_g_ = 124 °C [49]). Simultaneously, the energy gap decreases from 1.366 eV at 75 °C to 1.328 eV at 125 °C due to the presence of localized defect states near the edges of HOMO and LUMO bands, within the energy gap.

(III) 125–200 °C—for the PTB7-Th:ZY-4Cl blend film, we can observe the increase in Urbach energy, while the energy gap slightly decreases. The changes in Urbach energy for the blend with fullerene are not very significant, but the energy gap increases, compared to the value 1.328 eV at 125 °C to 1.358 eV at 175 °C. Annealing both blends up to 200 °C may lead to phase separation and cluster formation. More information can be seen in the AFM images of the blend film surfaces before and after annealing.

Absorption edge parameters obtained at room temperature, after the gradual annealing and then cooling to 25 °C, are seen in Figure 6 as the blue points. Thermally induced changes appear to be irreversible in both cases, as the measured values deviate from the initial parameters recorded at room temperature. For the PTB7-Th:ZY-4Cl blend, thermal treatment led to a decrease in Urbach energy and an increase in energy gap compared to the initial values, whereas the PTB7:PC70BM blend exhibited the opposite trend.

Based on the results of the thermo-optical investigations presented above, the optimal preliminary annealing temperature for solar cells with a PTB7-Th:ZY-4Cl active layer lies in the range of 75–100 °C, while for the fullerene-based blend, it is approximately 120 °C. At these respective temperatures, the lowest energy gap values are observed, and simultaneously, the Urbach energies are comparatively low.

### 3.2. Elipsometric Analysis

The spectroscopic ellipsometry is an optical reflective technique that measures the changes in light parameter polarization. The incident light beam is linearly polarized; after the reflection from the substrate, it has an elliptical polarization. Polarization parameters are the so-called ellipsometric angles *ψ* and *Δ*, the first of which describes the change in the polarization amplitude and the second its phase shift in the -*p* and -*s* electric field component vectors. *Ψ* and *Δ* angles are included in the main equation of ellipsometry, describing the complex reflectance *ρ*:

(4) $$\rho =e^{i\Delta }tan(\psi )$$

The reflection coefficient *ρ* can be determined theoretically for specific optical systems where the dielectric functions of the component layers are taken into account. In addition, *ρ* is a parameter that in fact strongly depends on the physical parameters of the material and experimental conditions, including film thickness, absorption, type of substrate, as well as the atmosphere in which the experiment is conducted (gas or vacuum). The *ρ* parameter can be determined by taking into consideration the dielectric functions of all layers included in the optical setup. The complex dielectric function is given by $\varepsilon =\varepsilon_{1}+i\varepsilon_{2}$, where ε_1_ is real, and ε_2_ is the imaginary component of this equation. The complex refractive index is given by $n=n_{0}+ik$, where the *n*_0_ is the real part of the function and *k*, so-called the extinction coefficient, is the imaginary part. The relation between dielectric function and refractive index is given by the relation $\varepsilon =n^{2}$.

Dispersions of optical (*n*, *k*) and dielectric (*ε*_1_, *ε*_2_) coefficients are determined for thin films of neat materials: PTB7-Th, ZY-4Cl, PC70BM and their blends PTB7-Th:PC70BM and PTB7:Th-ZY-4Cl, within the spectral range of 240–2500 nm, at room temperature, are presented in SI as Figures S2 and S3. The influence of annealing, at 100 and 200 °C, on the refractive index and extinction coefficient of thin films of ZY-4Cl, PTB7-Th and PC70BM is depicted in SI as Figures S4, S5 and S6, respectively.

**Figure 7:**
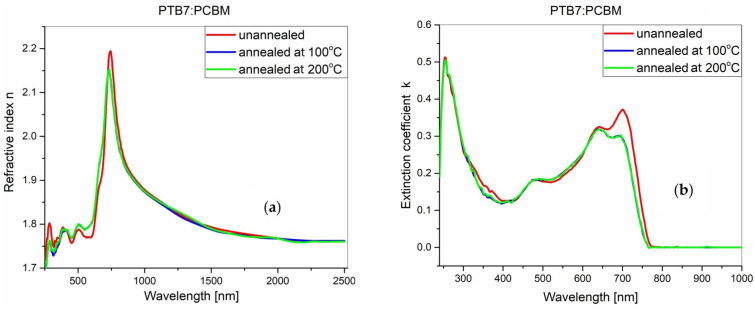
Effect of thermal annealing on the optical constants of the PTB7-Th:PC70BM active layer. (**a**) refractive index and (**b**) extension coefficient for the unannealed film and films annealed at 100 °C and 200 °C.

**Figure 8:**
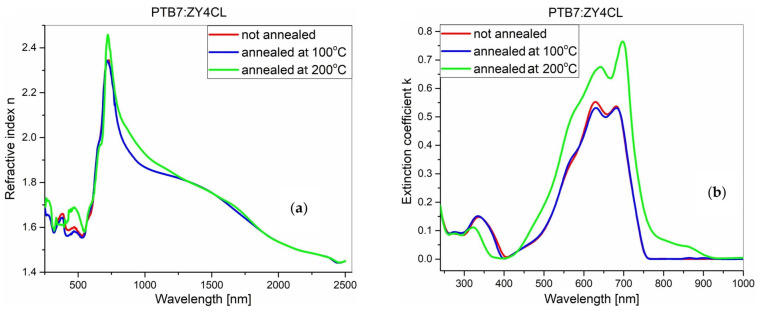
Effect of thermal annealing on the optical constants of the PTB7-Th:ZY4Cl active layer. (**a**) refractive index and (**b**) extinction coefficient for the unannealed film and films annealed at 100 °C and 200 °C.

These dependencies obtained for the blend films after annealing are presented in Figure 7 and Figure 8. No significant changes are observed in the refractive index spectra of the PTB7-Th:PC70BM blend film after annealing, unlike the dependencies of the extinction coefficient (see Figure 7). After annealing at 100 °C, the intensity of the extinction coefficient decreases, while after annealing at 200 °C, the intensity remains stable. The most likely explanation is the precipitation of the ordered PCBM phase on the film surface. As presented in our previous works [50,51], PCBM thin film has a glass transition temperature of approximately 110 °C, while its cold crystallization temperature is around 125 °C. Above this temperature, larger agglomerates of the ordered PCBM phase form readily, at the expense of film thickness and the volume of polymer, as similarly observed in [50]. The increasing agglomerates hinder the formation of PTB7-Th crystalline domains. At 200 °C, quite large ordered PCBM agglomerates are formed, and PTB7-Th cannot organize itself. Figure 8 shows the same set of parameters for the PTB7:ZY-4Cl blend film. After annealing at 100 °C, the spectral changes are slight, but after annealing at 200 °C, a significant increase in intensity and a broadening of the spectrum occur. In this case, the spectral broadening may be related to the scattering of light at the sample surface, where PTB7-Th domains are formed, which simultaneously contribute to the light absorption. According to the work of Yang et al. [52], ZY-4Cl has been shown to have a favorable effect on the creation of organized P3HT domains on the film surface. By varying the compound’s end groups, the authors found that the development of ordered P3HT domains is favored by the donor and acceptor’s limited miscibility. We think that PTB7-Th presents a comparable circumstance. The development of ordered PTB7-Th domains is further favored by ZY-4Cl’s high heat stability and slower rate of crystallization than PCBM.

### 3.3. Surface Morphology of Thin Films

**Figure 9:**
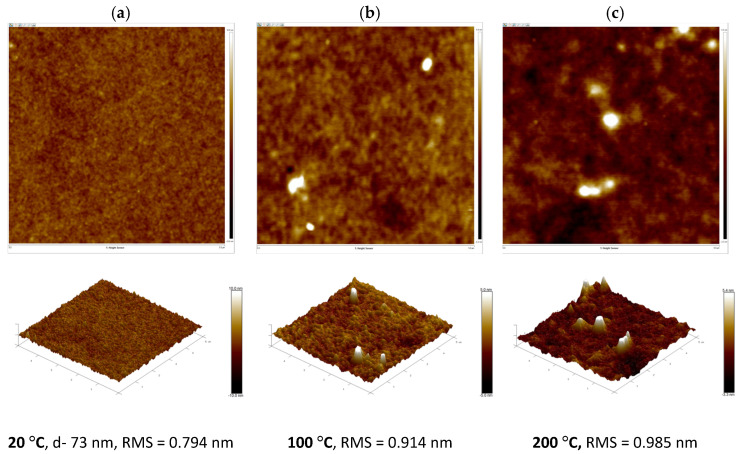
Two-dimensional (**top**) and three-dimensional (**bottom**) structures of PTB7-Th:PC70BM, area (5 × 5) μm: (**a**) before annealing (Height image: −10.0 to 10.0 nm), (**b**) after annealing at 100 °C (Height image: −5.0 to 5.0 nm) and (**c**) after annealing at 200 °C (Height image: −3.3 to 5.4 nm).

Surface morphology and thicknesses of thin films on glass substrates were measured using the AFM, which is operated in the soft tapping mode. For the PTB7-Th:PC70BM blend thin film, the root mean square (RMS) roughness was initially determined to be 0.794 nm. After annealing at 100 °C and 200 °C, the RMS values slightly increased to 0.91 nm and 0.985 nm, respectively. These results indicate minimal changes in surface morphology, with the film retaining a relatively smooth texture. However, following annealing at 200 °C (see Figure 9), the appearance of a few PCBM clusters becomes evident. A similar phenomenon was previously reported for the P3HT:PCBM blend, in which PCBM aggregation was observed post-annealing via optical microscopy, scanning electron microscopy (SEM), and atomic force microscopy (AFM) techniques [43].

**Figure 10:**
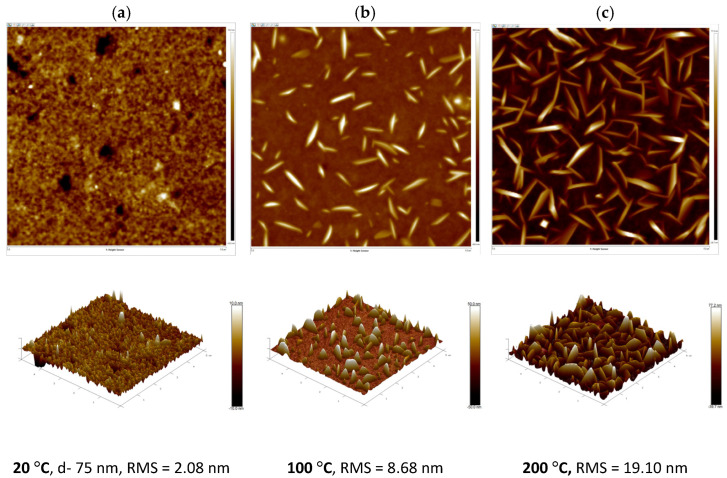
Two-dimensional (**top**) and three-dimensional (**bottom**) structures of PTB7-Th:ZY-4Cl, area (5 × 5) μm: (**a**) before annealing (Height image: −10.0 to 10.0 nm), (**b**) after annealing at 100 °C (Height image: −50.0 to 50.0 nm) and (**c**) after annealing at 200 °C (Height image: −39.7 to 77.2 nm).

Quite different results were obtained for the blend with a non-fullerene acceptor. The thin film of the PTB7-Th:ZY-4Cl blend was annealed at 100 °C and 200 °C, and changes observed on the surface morphology were spectacular (see Figure 10b,c). As the annealing temperature increased from room temperature to 100 °C and 200 °C, the phase separation occurred, and the RMS value clearly increased. To compare the role of individual constituents of blends, the morphology of thin films of pure PTB7-Th and ZY-4Cl was also investigated before and after annealing (see Supplementary Information, Figures S7 and S8). To know what type of material (donor, acceptor or blend) can be inside these crystallites, we decided to use nano-IR investigations, but first, the ATR-FTIR spectra were obtained to choose the suitable wavelengths of IR absorption.

Table 1 lists the parameters obtained from AFM measurements for the PTB7-Th:PC70BM and PTB7-Th:ZY4Cl blend films (see Figure 9 and Figure 10) as well as for pristine PTB7-Th and ZY4Cl films (see Figures S7 and S8 in SI), measured before and after annealing at 100 °C and 200 °C.

### 3.4. ATR-FTIR and Nano-IR Results of Measurements

**Figure 11:**
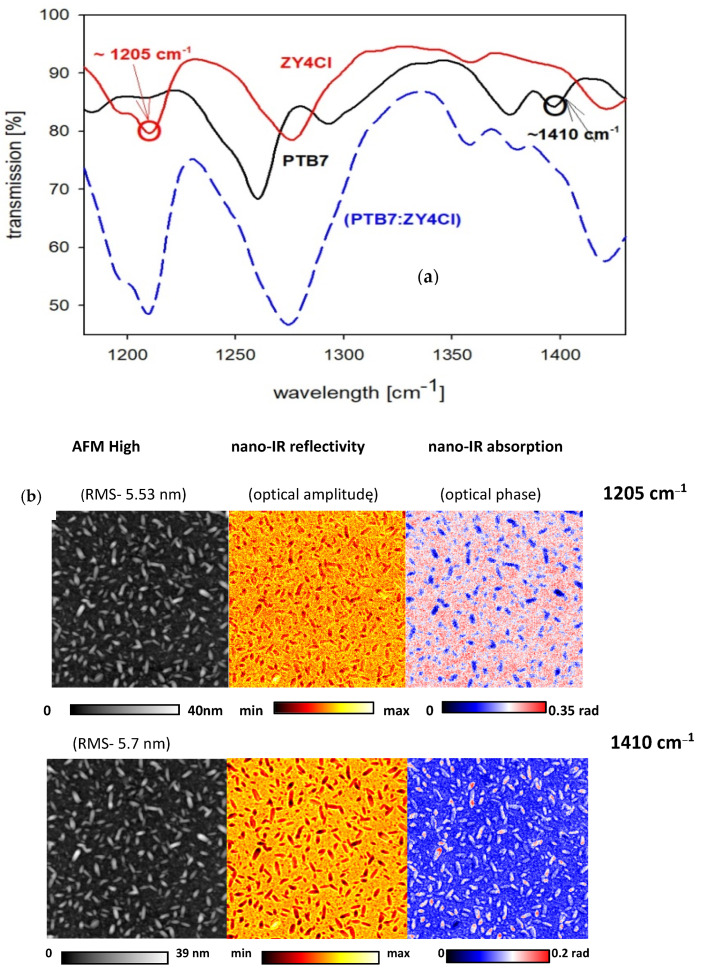
(**a**) ATR-FTIR spectra of PTB7-Th, ZY-4Cl and blend films, within the range (1180–1430) cm^−1^; (**b**) s-SNOM images, recorded at 1205 cm^−1^ and 1410 cm^−1^ of the PTB7-Th:ZY4Cl blend film, after annealing at 100 °C; scan area (5 × 5) µm and (200 × 200) pix, with 10 ms/pix.

ATR-FTIR spectra within the wide spectral range (400–4000 cm^−1^) for blend films based on both types of acceptor are presented in Supplementary Information (SI) in Figure S9d,e, while the spectra of pure polymers, the PTB7-Th, ZY-4Cl and PC70BM films, are shown in the SI as Figure S9a–c. On the basis of ATR-FTIR spectra of the PTB7-Th and ZY-4Cl films, two absorption wavelengths, 1410 cm^−1^ and 1205 cm^−1^, respectively (see Figure 11a), were chosen to further nano-IR experiments. ATR-FTIR spectra of PTB7-Th and ZY-4Cl films together with the spectra of the blend film are presented in Figure 11a, within the chosen spectral range (1180–1430) cm^−1^.

The AFM images show the presence of needle-shaped nano-crystallites on the surface of the PTB7-Th:ZY-4Cl blend films after annealing at 100 °C and 200 °C (see Figure 10a,b). Then we examined the blend film, deposited on a silicon substrate, before and after annealing at 100 °C, by the nano-IR experiments, to explain the chemical nature of these nano-crystallites on the film surface. Nano-IR sequential imaging of the PTB7-Th:ZY-4Cl blend film surface at specific IR bands (1205 cm^−1^ and 1410 cm^−1^) can indicate the composition of these nano-crystallites, by the presence of absorption contrasts between the different components within the blend. S-SNOM (Scattering-Type Scanning Near-field Optical Microscopy) technique delivers materials characteristic maps of chemical and optical properties of the sample surface, simultaneously with the topographic (AFM) imaging. Figure 11b presents images obtained for the PTB7-Th:ZY-4Cl film surface after annealing at 100 °C, under the illumination light 1205 cm^−1^ and 1410 cm^−1^, while the images of the film surface before annealing are shown in SI as Figure S10. The scan area was 5 × 5 μm with a resolution of 200 × 200 pix and a scan time of 10 ms/pix. These frequencies were chosen on the basis of ATR-FTIR spectra, as it is seen in Figure 11a.

The red color on nano-IR-absorption images (seen in Figure 11b) corresponds to the region of higher absorption, which may indicate that PTB7-Th predominates within these needle-shaped nano-crystallites, seen after annealing at 100 °C, on the surface of the PTB7-Th:ZY4Cl blend film. The above-presented results demonstrate that this advanced IR-s-SNOM technique is ideally suited for monitoring polymorphism and phase coexistence in highly ordered organic films, like in the work [53], and to map defective molecular crystallites on the nanoscale, as in [54].

### 3.5. J-V Characteristics of Fullerene and Non-Fullerene-Based BHJ Solar Cells

**Figure 12:**
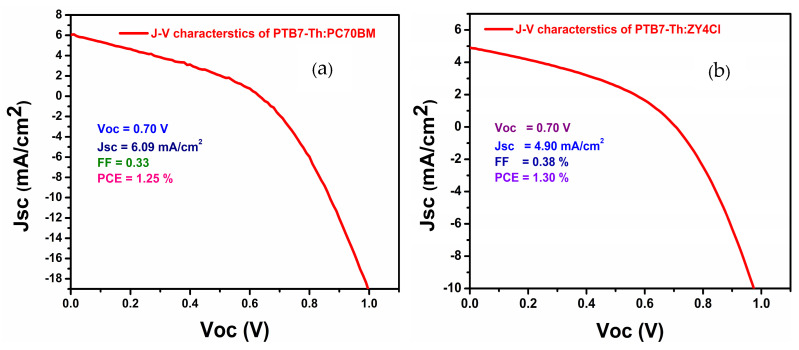
Current density–voltage (J-V) characteristics of the best performing devices based on (**a**) the fullerene type acceptor PC70BM and (**b**) the non-fullerene type acceptor ZY-4Cl, measured under standard illumination conditions.

To correlate and summarize the results presented above, the BHJ solar cells were fabricated and examined. Three photovoltaic devices were fabricated for each fullerene and non-fullerene-based active layer, with an active area of 0.04 cm^2^. The average photovoltaic (PV) parameters along with their standard deviations were calculated and summarized in Table 2, while Figure 12 presents the current density–voltage (J-V) characteristics of the best photovoltaic devices. The solar cells were fabricated under the ambient conditions, i.e., without the use of a glovebox and without employing solid and solvent additives. According to the above results, the blend films were annealed at 120 °C and about 85 °C, for fullerene and non-fullerene acceptor, respectively. Under the standard conditions, i.e., AM 1.5G, irradiance 1000 W/m^2^, the non-fullerene-based device achieved a PCE of 1.30% while the fullerene-based device exhibited a PCE of 1.25%. Fabrication under ambient conditions introduces oxygen and moisture traps at the donor–acceptor interface, which reduce carrier lifetime and increase recombination. Controlled processing conditions, along with the optimized post annealing temperatures, can lead to enhanced (PV) performance.

## 4. Summary and Conclusions

In conclusion, this study reported on the influence of acceptor type (fullerene or non-fullerene) on the thermo-optical and morphological properties of PTB7-Th:PC70BM and PTB7-Th:ZY-4Cl blend films, used as active layers in BHJ polymer solar cells.

The optical properties of these blend films were examined during stepwise annealing, using in situ UV-Vis-NIR(*T*) absorption measurements. These results allowed us to present and discuss the changes in absorption edge parameters, i.e., energy gaps (*E*_G_) and the Urbach energy (*E*_U_), at elevated temperatures, depending on the type of acceptor used in the investigated blend films. Analysis of temperature dependences of these parameters allowed us to divide the annealing process into the main successive stages, where the different temperature-induced structural changes were observed. Changes in refractive index and extinction coefficient, obtained from ellipsometric measurements, were also presented for PTB7-Th:PC70BM and PTB7-Th:ZY-4Cl blend films. To explain the different behavior of these two types of blend films at higher temperatures, the same investigations were performed for the pristine PTB7-Th, ZY-4Cl and PC70BM films. In the case of a blend with non-fullerene acceptor ZY-4Cl, this compound decides on the blend behavior at higher temperatures, while for the blend with the fullerene acceptor, the dominant role is played by the donor polymer PTB7-Th. This is attributed to the high thermal stability of PC70BM, for which the absorption edge parameters are almost constant during annealing up to 200 °C. Based on the thermo-optical investigations, the optimal preliminary annealing temperature for solar cells with PTB7:ZY-4Cl active layer was determined to be approximately 75–100 °C, while for the blend with fullerene acceptor, it is about 120 °C. At these temperatures, reduced energy gap values were observed while the Urbach energies remained relatively low. Moreover, AFM images of the PTB7-Th:PC70BM blend film revealed a relatively smooth surface, while PTB7-Th:ZY-4Cl blend films exhibited needle-shaped crystallites on the surface, after annealing at 100 °C and 200 °C. Nano-IR absorption images, obtained using the s-SNOM technique, allowed the presentation of materials characteristic maps of chemical and optical properties of the film surface, and to conclude that polymer PTB7-Th predominates within these needle-shaped crystallites. Finally, for both types of blends, used as active layers in BHJ organic solar cells, a photovoltaic effect was observed, and PV parameters were determined.

The presented thermo-optical (in situ) and surface morphology (AFM and s-SNOM) investigations confirm the critical role of acceptor type in determining the thermal and structural stability of the BHJ active layer, particularly at higher temperatures. The novelty of this work lies in the comprehensive experimental comparison of blend films under controlled annealing, providing new insight into their higher temperature behavior. Moreover, the PTB7-Th:ZY-4Cl blend represents a relatively new studied system for BHJ organic solar cells. The applied experimental approach offers a useful framework for investigating thin films, to choose the best materials and post-deposition strategy to improve the photovoltaic device performance.

## Figures and Tables

**Table 1:** Thicknesses and RMS roughness values of blends and pristine films measured by AFM.

Film	Thickness [nm] at 20 °C	RMS [nm] at 20 °C	RMS [nm] at 100 °C	RMS [nm] at 200 °C
PTB7-Th:PC70BM	63.00	0.79	0.914	0.98
PTB7-Th:ZY-4Cl	75.00	2.08	8.68	19.10
PTB7-Th	81.00	1.10	0.97	1.10
ZY-4Cl	43.00	21.00	18.60	14.90

**Table 2:** Average photovoltaic parameters of BHJ solar cells under standard illumination for fullerene and non-fullerene-based devices.

Classification	Voc [V]	Jsc [mA/cm^2^]	FF	PCE [%]
PTB7-Th:ZY-4Cl	0.686 ± 0.013	4.76 ± 0.14	0.36 ± 0.015	1.19 ± 0.10
PTB7-Th:PC70BM	0.65 ± 0.044	6.06 ± 0.031	0.32 ± 0.01	1.22 ± 0.027

## Data Availability

The original contributions presented in this study are included in the article/Supplementary Material. Further inquiries can be directed to the corresponding authors.

## Supplementary Materials

The following supporting information can be downloaded at: https://www.mdpi.com/article/10.3390/polym18020280/s1, Figure S1: Absorption edge parameters of: (a) ZY-4Cl and (b) PTB7-Th films, obtained before and after annealing at 100, 150, 200 °C. Figure S2: Refractive index (a) and extinction coefficients (b) of PCBM, PTB7-Th, ZY-4Cl and their blends. Figure S3: Real (a) and imaginary (b) parts of dielectric function of thin films of PCBM, PTB7-Th, ZY-4Cl and their blends. Figure S4: Influence of annealing ZY4Cl film on: (a) refractive index and (b) extinction coefficient. Figure S5: Influence of annealing PTB7-Th film on (a) refractive index and (b) extinction coefficient. Figure S6: Influence of annealing PCBM film on (a) refractive index and (b) extinction coefficient. Figure S7: 3D and 2D AFM images of the structure of PTB7-Th film surface area, 5 × 5 μm (a) before annealing: (RMS = 1.10 nm) thickness (81 nm) (Height image: −10 to 10 nm) (b) after annealing (100 °C): (RMS = 0.97 nm) (Height image: −5.0 to 5.0 nm) (c) after annealing (200 °C): (RMS =1.10 nm) (Height image: −6.3 to 3.6 nm). Figure S8: 3D and 2D AFM images of ZY-4Cl film surface area, 5 × 5 μm (a) before annealing: (RMS = 21 nm) thickness (43 nm) (Height image: −46.2 to 45.8 nm) (b) after annealing (100 °C): (RMS = 18.6 nm) (−45.0 to 38.2) (c) after annealing (200 °C): (RMS = 14.9 nm) (Height image: −41.2 to 48.4 nm). Figure S9: ATR-FTIR spectra of thin films of PTB7-Th, ZY4Cl, PCBM and blends. Figure S10: s-SNOM images (AFM high, nano-IR reflectivity, nano-IR absorption) recorded at 1205 cm^−1^ and 1410 cm^−1^ of PTB7-Th:ZY4Cl blend film, before annealing (at room temperature); Scan area (5 × 5) µm and (200 × 200) pix, 10 ms/pix. Table S1: Thicknesses and RMS values of blend and pristine films, obtained from AFM measurements.
