# Supplementary material for: The Effect of Thermal Annealing on Optical Properties and Surface Morphology of a Polymer: Fullerene- and Non-Fullerene-Blend Films Used in Organic Solar Cells
Source: Polymers (Basel). 2026 Jan 20;18(2):280. doi: 10.3390/polym18020280 (PMC12846255; doi:10.3390/polym18020280)
Supplement: Supplementary file 1 [file polymers-18-00280-s001.zip › polymers-4056807-supplementary.pdf]

Supplementary Data

Article

The Effect of Thermal Annealing on Optical Properties and Surface Morphology of a Polymer: Fullerene- and Non-Fullerene Blend-Films Used in Organic Solar Cells

Bożena Jarząbek [1,*], Muhammad Raheel Khan [1,2,*], Barbara Hajduk [1], Andrzej Marcinkowski [1], Paweł Chaber [1], Adrian Cernescu [3] and Yasin C. Durmaz [3]

[1] Centre of Polymer and Carbon Materials, Polish Academy of Sciences, Sklodowskai-Curie 34 Str., 41-819 Zabrze, Poland; bhajduk@cmpw-pan.pl (B.H.); amarcinkowski@cmpw-pan.pl (A.M.); pchaber@cmpw-pan.pl (P.C.)
[2] Joint Doctoral School, Silesian University of Technology, Akademicka 2A, 44-100 Gliwice, Poland
[3] Attocube systems AG, Eglfinger Weg 2, 85540 Munich, Germany; adrian.cernescu@attocube.com (A.C.); yasin.durmaz@attocube.com (Y.C.D.)
* Correspondence: bjarzabek@cmpw-pan.pl (B.J.); mukhan@polsl.pl (M.R.K.)

Within the framework of this Supplementary Information, we present the influence of annealing on thin films of PTB7-Th, ZY-4Cl, PC70BM using absorption, elipsometric and AFM studies. Moreover, the ATR-FTIR spectra of all neat and blend thin films and s-SNOM images of PTB7-Th:ZY-4Cl film before annealing are shown.

Contents

S1. Absorption edge parameters of PTB7-Th and ZY-4Cl films after annealing

S2. Elipsometric results:

S2-1.Refractive index and extinction coefficient at room temperature

S2-2. Influence of annealing on PTB7-Th, ZY-4Cl and PC70BM films

S3. AFM images of thin films surfaces of PTB7-Th and ZY-4Cl - the influence of annealing

S4. ATR-FTIR spectra of thin films of polymers and blends

S5. *s-SNOM images of PTB7-Th:ZY4Cl film before annealing*

S1. Absorption edge parameters.

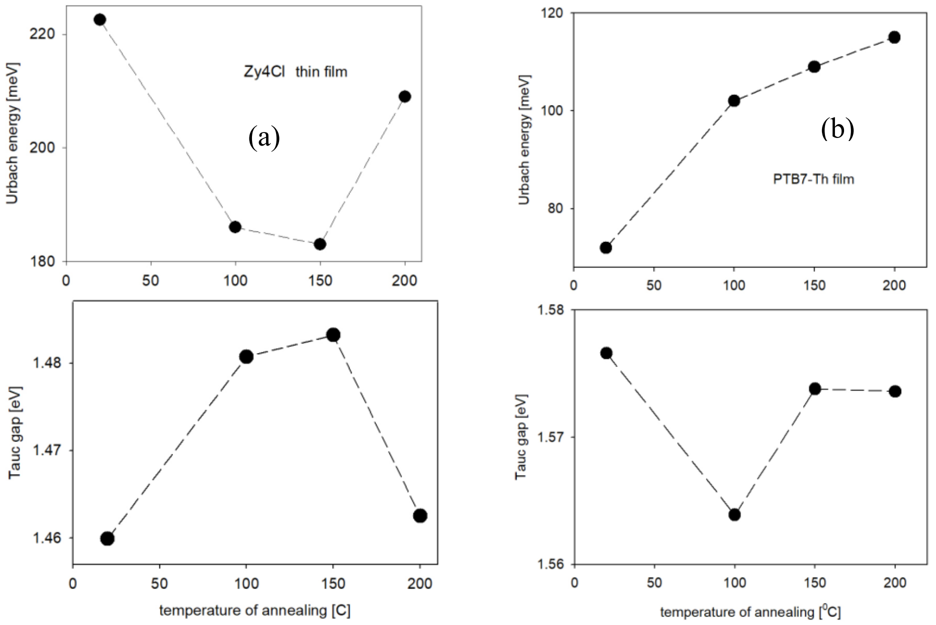

Figure S1. Absorption edge parameters of: (a) ZY-4Cl and (b) PTB7-Th films, obtained before and after annealing at 100, 150, 200 °C.

In our present studies we investigated the absorption coefficient spectra, edge parameters at room temperature and after annealing at 100, 150, 200 °C respectively, for the neat films of ZY4Cl (acceptor) and PTB7-Th (donor) what is depicted in **Figure S1,** to discuss their role within blends, during annealing. In the case of fullerene film, it was presented earlier in [43] that PCBM films are stable during annealing and absorption edge parameters determined within the range of 25-200 °C are almost invariable.

These dependences, presented in **Figure S1** can be compare with the **Figure 5** and it is seen that ZY-4Cl plays important role in the PTB7-Th:ZY-4CL blend, while PTB7-Th determines the behavior of PTB7-Th:PC70BM blend, during annealing.

S2. Elipsometric results

S2-1.Refractive index and extinction coefficient at room temperature

The values of the optical coefficients: refractive index (*n)* and extinction coefficient (*k*) for PC70BM, PTB7-Th, ZY-4CL and their blends obtained at room temperature, within the 240-2500 nm spectral range are presented in **Figures. S2 (a, b)** while the real (ε_1) and imaginary (ε_2) parts of dielectric function spectral dependences are shown in **Figures S3 (a, b).**

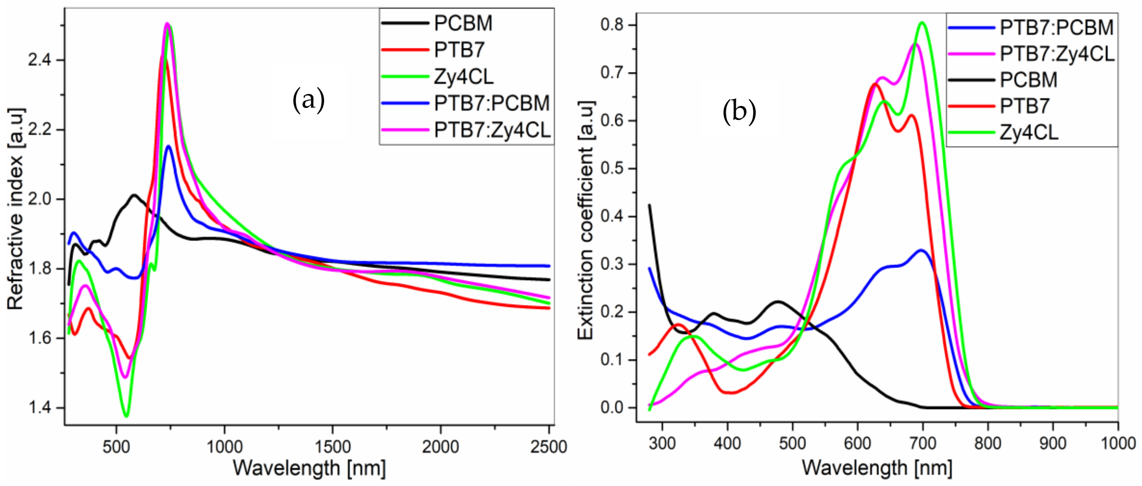

Figure S2. Refractive index (a) and extinction coefficients (b) of PCBM, PTB7-Th, ZY-4Cl and their blends.

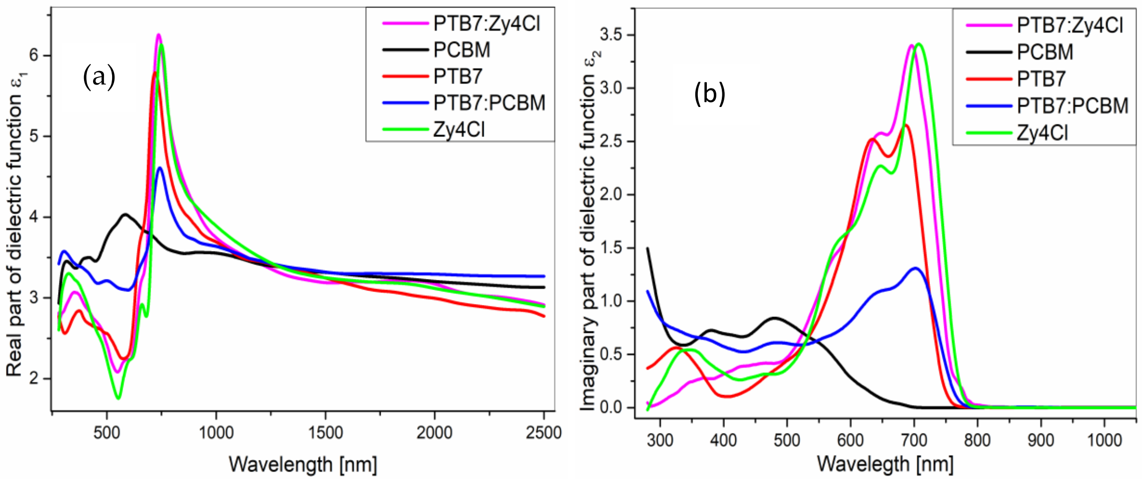

Figure S3. Real (a) and imaginary (b) parts of dielectric function of thin films of PCBM, PTB7-Th, ZY-4Cl and their blends.

S2-2. Influence of annealing

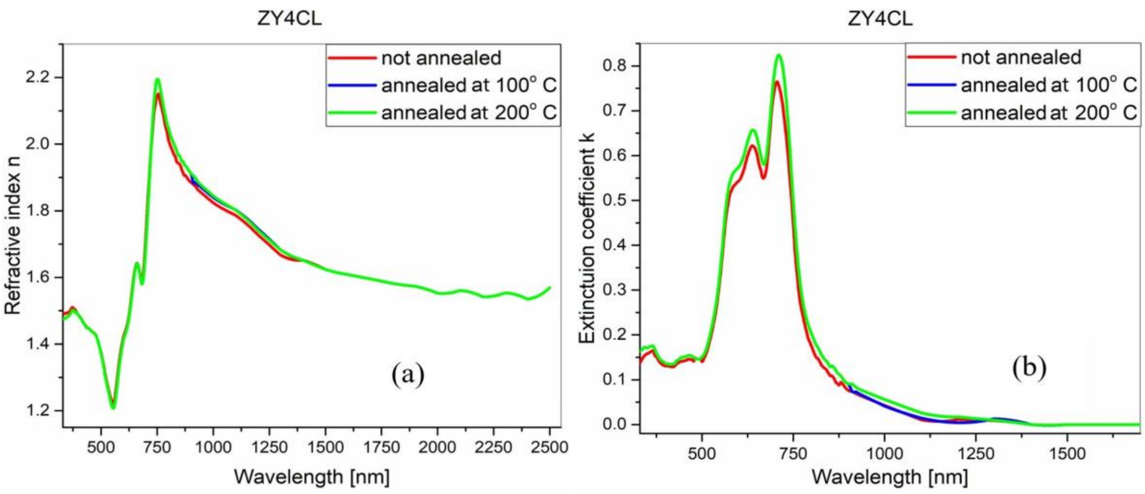

Figure S4. Influence of annealing ZY4Cl film on: (a) refractive index and (b) extinction coefficient.

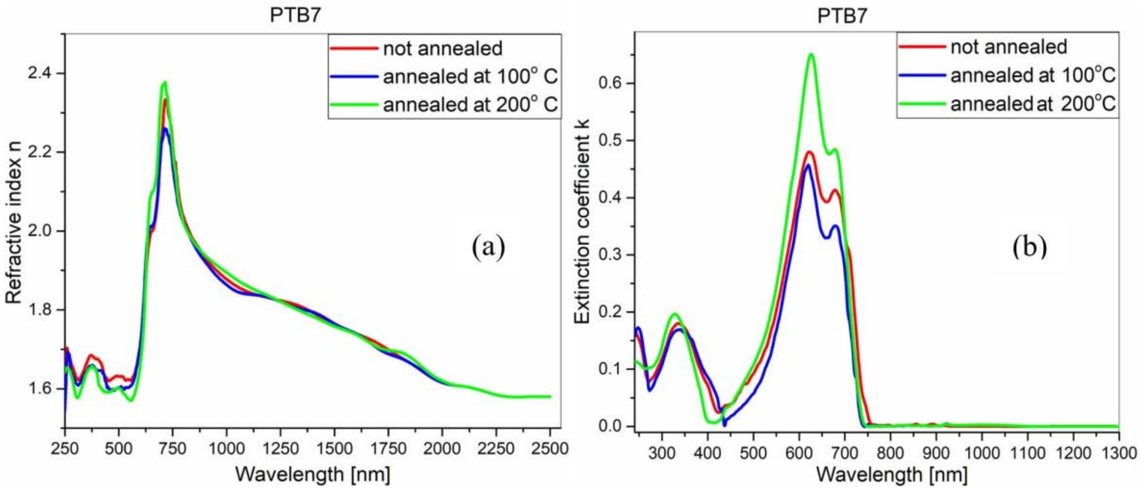

Figure S5. Influence of annealing PTB7-Th film on (a) refractive index and(b) extinction coefficient.

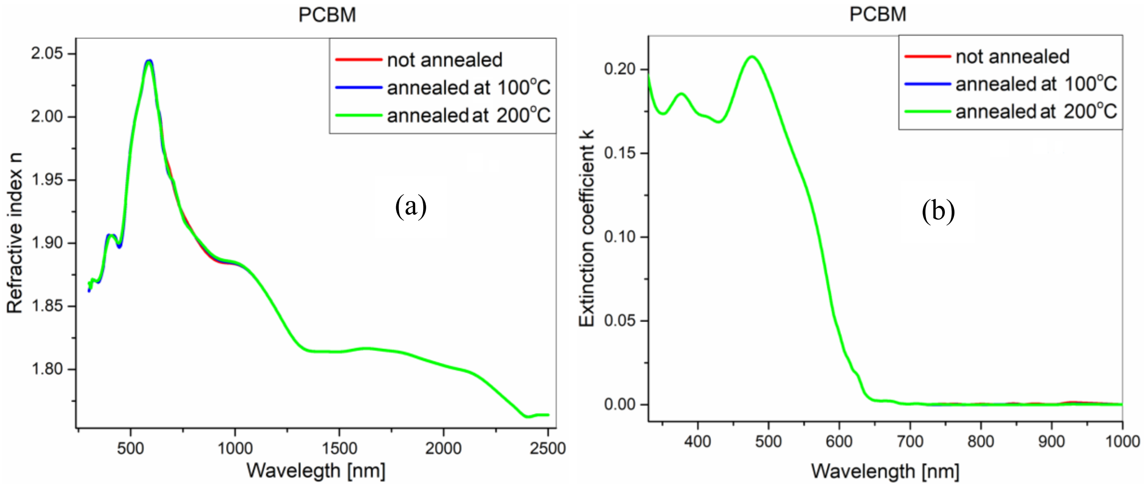

Figure S6. Influence of annealing PCBM film on (a) refractive index and (b) extinction coefficient.

These dependences, presented in **Figures S4**, **S5** and **S6** indicate that after annealing at 200 °C the changes of absorption (extinction coefficient) are more seen for PTB7-Th and less for ZY-4Cl . Results obtained for PCBM once more confirmed well known thermal stability of this type of fullerene film. In the case of blends (see **Figure 7** and **Figure 8** in the main text) the changes are clearly seen for PTB7-Th:ZY-4Cl while for blend PTB7-Th:PCBM (**Figure 7**) annealing caused structural order and absorption after annealing (at 100 and 200 °C) was almost the same, smaller than in room temperature.

S3. AFM images of thin films surfaces of PTB7-Th and ZY4Cl films - the influence of annealing

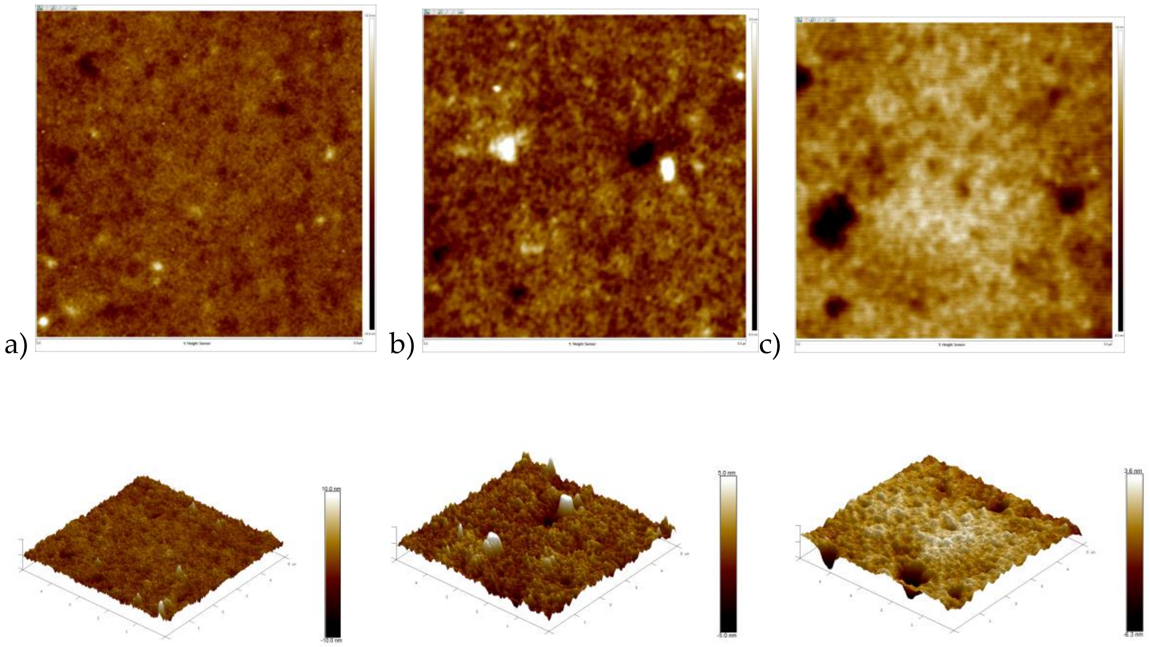

Figure S7 : 3D and 2D AFM images of the structure of PTB7-Th film surface area, 5x5 μm (a) before annealing: (RMS = 1.10 nm) thickness (81 nm) (Height image: -10 to 10 nm) (b) after annealing (100 °C): (RMS = 0.97 nm) (Height image: -5.0 to 5.0 nm) (c) after annealing (200 °C): (RMS =1.10 nm) (Height image: -6.3 to 3.6 nm).

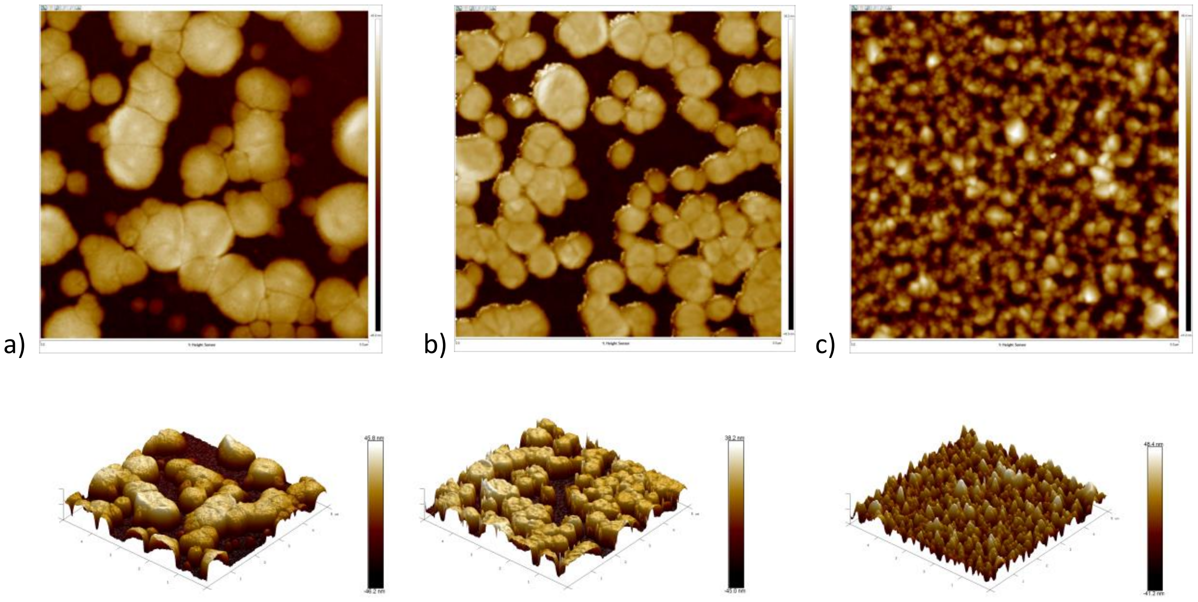

Figure S8 : 3D and 2D AFM images of ZY-4Cl film surface area, 5x5 μm (a) before annealing: RMS =21 nm) thickness (43 nm) (Height image: -46.2 to 45.8 nm) (b) after annealing (100 °C): (RMS =18.6 nm) (Height image: -45.0 to 38.2) (c) after annealing (200 °C): (RMS =14.9 nm) (Height image: -41.2 to 48.4 nm).

Comparing **Figures S7** and **S8** it is clearly seen the different character of PTB7-Th and ZY-4Cl surface and their behavior after annealing. The surface of PTB7-Th film is rather smooth, also after annealing at 200 °C (RMS ≅ 1 nm). In the case of ZY4-Cl film the surface has granular character and after heat treatment these granulates are smaller and RMS decrease from 21 nm at room temperature to about 15 nm after annealing at 200 °C. In **Table S1** are gathered the results for PTB7-Th and ZY-4Cl films together with these data obtained for blends (AFM images of blend films are presented in the main part of text).

Table S1. Thicknesses and RMS values of blend and pristine films, obtained from AFM measurements

Film	Thickness [nm] at 20 °C	RMS [nm] at 20 °C	RMS [nm] at 100 °C	RMS [nm] at 200 °C
PTB7-Th:PC70BM	63.00	0.79	0.914	0.98
PTB7-Th: ZY-4Cl	75.00	2.08	8.68	19.10
PTB7-Th	81.00	1.10	0.97	1.10
ZY4CL	43.00	21.00	18.60	14.90

S4. ATR-FTIR spectra of thin films of polymers and blends

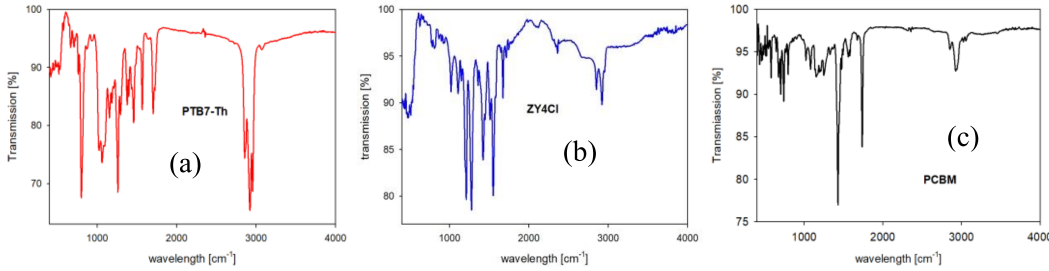

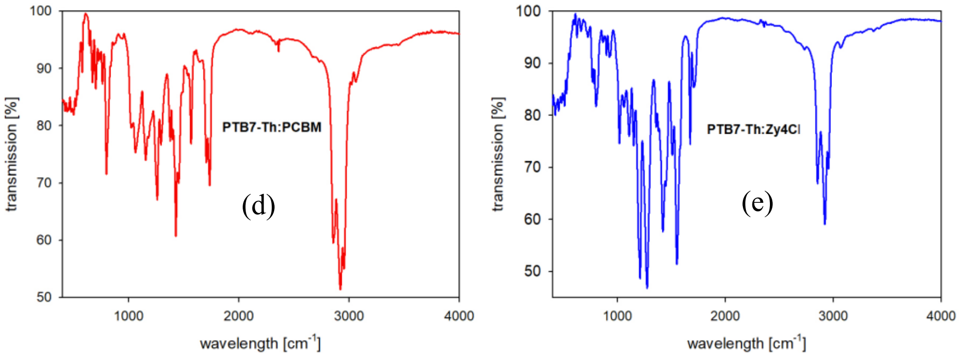

Figure S9. ATR-FTIR spectra of thin films of PTB7-Th, ZY4Cl, PCBM and blends.

S5. ***s-SNOM images of PTB7-Th:ZY4Cl blend film before annealing***

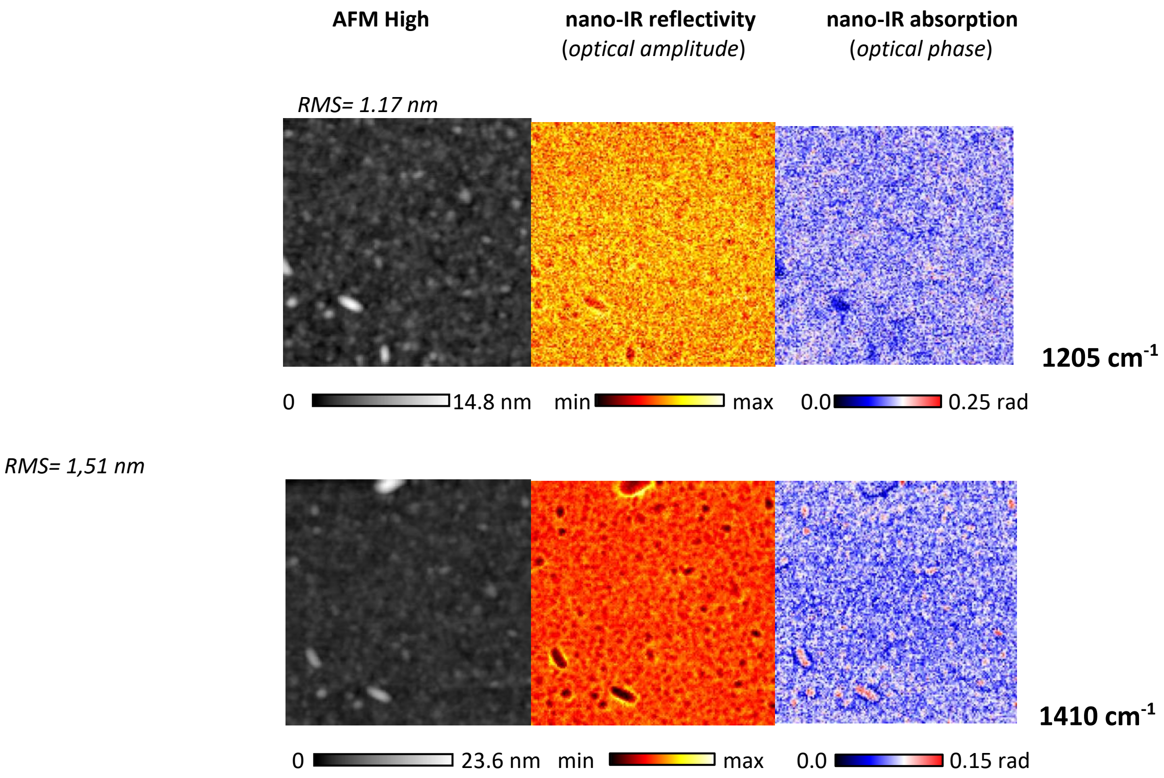

Figure S10. s-SNOM images (AFM high, nano-IR reflectivity, nano-IR absorption) recorded at 1205 cm^{-1} and 1410 cm^{-1} of PTB7-Th:ZY4Cl blend film, before annealing (at room temperature); Scan area (5x5) μm and (200x200) pix, 10 ms/pix.

We can see no changes, on the nano-IR absorption images, depend on these two wavelengths of light, what confirms uniformity of the blend film surface.

References

[43] B. Jarząbek, P. Nitschke , B. Hajduk , M. Domanski , H. Bednarski, *In situ* thermo-optical studies of polymer: fullerene blend films Polymer Testing 88 (2020) 106573, https://doi.org/10.1016/j.polymertesting.2020.106573.
